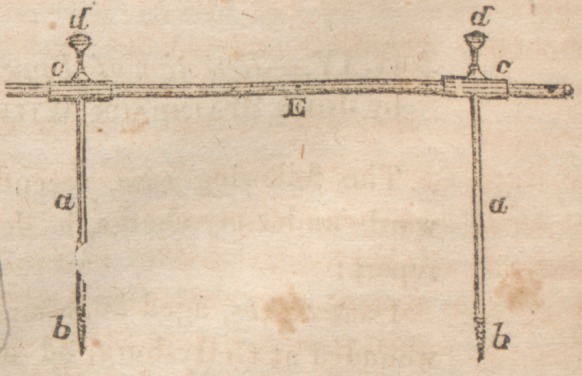# New Method of Treating Ununited Fracture of Long Bones

**Published:** 1864-04

**Authors:** James Bolton

**Affiliations:** Surg. P. A. C. S.


**Art. III.**—*New method of treating Ununited Fracture of Long Bones.* By James Bolton, Surg. P. A. C. S.

My observations on cases of gun-shot wounds of the thigh, with fracture of the bone, have impressed me strongly with the unsatisfactory character of the ordinary methods of treating them. The difficulties encountered are: 1st. The great mortality in this class of injuries; 2d. The frequent movement of the limb for the purpose of cleansing it, producing severe and exhausting suffering, and interfering with the union of the bone; 3d. The splintered condition of the fractured ends of the bone interfering with accurate adjustment; 4th. This condition, together with the exposed state of the fractured ends, favoring necrosis and exfoliation; 5th. The long period usually required for union prohibiting the continuous use of extension and counter-extension; 6th The contraction of the muscles during this long period causing shortening and angular displacement; 7th. Bed-sores produced by long confinement of the patient in one position. The hazards of this injury are fearful. Amputation is only an exchange of risk of death by exhaustion, for risk of death by shock. If the limb be saved, it will almost certainly be shortened, and frequently be misshapen; and this may happen to such an extent that the limb may be an incumbrance. In view of these difficulties, I respectfully ask attention to a new method by which I propose, as far as practicable, to obviate them. The indications are to secure co-aptation, adaptation and quietude. The method by which I propose to fulfil these indications is explained in the following sketch of a case which occurred in my practice in December, 1853: An Irish laborer had ununited fracture of the femur. The ends overlapped more than two inches, and were kept asunder by intervening soft tissue. The only method which seemed applicable was that of sawing off the ends of the fragments and wiring them. The feeble condition of the patient and the great mortality attendant upon this operation determined me to endeavor to devise some other method of treatment. In the meantime, efforts were made, which proved futile, to co-apt and fix the fractured ends. I then employed the following method: Two steel rods, *a*, three inches in length, were each cut at one end into a screw, *b*, three-eighths of an inch long. To the other end was attached, at right angles, a hollow cylinder, *c*, having on its upper surface a screw, *d*.

A steel rod, *e*, five inches long, completed the instrument. The patient was anæsthetized, and powerful extension was used until the limb was brought to the proper length. The point of a scalpel was then thrust down to each fragment, one inch from the extremity, and a hole was drilled in each as far as the medullary canal. In these holes the rods, *a*, were screwed. The ends of the bone were then adjusted by pressure upon one rod and by traction upon the other. When adjustment was complete, the cylinders, *c*, were in a line with each other. The rod, *e*, was then passed through them and secured by the screws, *d*. This rod was outside of the integuments, and was of necessity parallel with the shaft of the bone. The only motion then possible was a rotary one upon the screws. This was prevented by straight splints upon opposite sides of the thigh. The same thing might have been accomplished by applying another instrument at right angles to the first. The limb was placed upon a pillow. No exhaustion to the patient was produced, and he stated that he felt more comfortable than he did before the instrument was applied. It was retained upon the limb twenty-one days. It was then removed, and straight splints alone used. Eight days afterwards the patient died from a cause unconnected with the use of the instrument. The lower fragment was found necrosed at the upper extremity, evidently from violence done in tearing it from its adhesions, whereby a portion of the periosteum was stripped off. The points into which the rods were screwed were sound, and the ends were united by a thick cartilaginous bridge. Besides the advantages stated, the instrument produced another beneficial effect, viz: stimulating the bone to throw out uniting osseous matter, as in the method adopted by Dieffenbach, who drilled the ends of the bones, and drove in ivory pegs. Malgaigne adopted a method somewhat similar to mine. He passed under the limb a strap attached to a steel bow, placed over the limb, having a long screw passing through the centre of the bow. This screw, having a pointed extremity, he brought to bear upon a displaced fragment, and, by turns of the screw, forced the fragment into position; but the fragments were then in a state of almost uncontrolled mobility.

The instrument is evidently applicable to other long bones, and to the lower jaw.

NOTE.—It may be objected that by this method a simple fracture may be converted into a compound one; but a careful consideration will lead to the conviction that this result ought not to occur. In the case of gun-shot wound the fracture is already compound.

## Figures and Tables

**Figure f1:**